# Urdu translation and validation of premature ejaculation diagnostic tool (PEDT)

**DOI:** 10.12669/pjms.36.6.2405

**Published:** 2020

**Authors:** Muhibullah Bangash, Wajahat Aziz, Mohammad Shoaib, M Hammad Ather

**Affiliations:** 1 Muhibullah Bangash, MBBS. Urology Resident. (Year-VI), Urology Section, Aga Khan University Hospital, Karachi, Pakistan; 2 Wajahat Aziz, MBBS, FCPS (Urol), Senior Instructor, Urology Section, Aga Khan University Hospital, Karachi, Pakistan; 3 Mohammad Shoaib, MBBS. Urology Resident. (Year-V), Urology Section, Aga Khan University Hospital, Karachi, Pakistan; 4 M Hammad Ather, MBBS, FCPS (Urol), FRCS, FEBU. Prof and Section Head of Urology, Urology Section, Aga Khan University Hospital, Karachi, Pakistan

**Keywords:** Premature ejaculation (PE), Premature Ejaculation Diagnostic Tool (PEDT), Intra-vaginal ejaculatory latency time (IELT)

## Abstract

**Objective:**

To validate an Urdu translation of premature ejaculation diagnostic tool (PEDT) by analyzing the association of this diagnostic tool with the clinical diagnosis of premature ejaculation (PE) and intravaginal ejaculatory latency time (IELT).

**Methods:**

This cross-sectional study was conducted at the urology section of the Aga Khan University Hospital, Karachi, for six months duration, from July 2018 to December 2018. In our study 108 subjects, aged 20 to 50 years, who were in a stable sexual relationship (heterosexual) for a minimum duration of six months, were asked to fill the Urdu version of PEDT, 61 with PE and 47 without PE.

**Results:**

The two groups matched for mean age, duration of relationship and education level. The duration of 1.2 (±0.5) minutes was the mean self-estimated IELT in the PE group and 3.7±0.9 minutes in patients without PE. There was a significant negative correlation of 0.6 (p-value <0.001) between the PEDT score and self-estimated IELT. The test-retest reliability for each item was found to be significant for each individual item (≥ 0.84, p-value <0.001) and 0.94 was the correlation coefficients of the total score, showing an excellent test-retest reliability. 0.93 was the Cronbach’s alpha score (95% Confidence interval = 0.905 - 0.948) indicating a significant internal consistency in the Urdu version of PEDT.

**Conclusions:**

The Urdu version of PEDT is a valid tool to define and quantify PE objectively, with adequate internal consistency. This version of PEDT has a good negative correlation with self-estimated IELT and excellent correlation with clinical PE.

## INTRODUCTION

Premature ejaculation (PE) is an ejaculatory disorder with a highly variable estimated prevalence which is partly explained by the lack of standardized definitions and diagnostic tools.^1^ There are various definitions of premature ejaculation in literature. In the Diagnostic and Statistical Manual of Mental Disorders-IV-Text Revision (DSM-IV-TR), PE was defined according to patient description and clinician assessment of a few other factors.^2^ The International Society for Sexual Medicine (ISSM) adopted the first evidence-based objective definition of PE.^3^ Premature ejaculation (lifelong and acquired) is a male sexual dysfunction with following features:

1. Ejaculation that always occurs before or within about one minute of vaginal penetration (lifelong PE) or a clinically reduced in latency time, to about three minutes or less (acquired PE).
2. Unable to delay ejaculation on all or nearly all-vaginal penetrations.
3. Negative personal consequences, such as distress, bother, frustration, and/or the avoidance of sexual intimacy.

Several questionnaires were developed to objectively diagnose premature ejaculation. Two of the most commonly used tools to diagnose PE, based upon patient-reported outcomes are Arabic Index of Premature Ejaculation (AIPE) and Premature Ejaculation Diagnostic Tool (PEDT). PEDT has been the most widely used tool in many countries and it is a brief questionnaire comprising of five items.^4^ PEDT score of more than 11 indicates PE diagnosis, a score of 9 or 10 implies a probable PE, while a score of < 8 indicates no PE. PEDT has standardized the diagnosis of PE and has already been translated and validated in various languages. However, an Urdu translation and validation of PEDT does not exist. Urdu is the language widely spoken and understood in Pakistan with a population of around 217 million (worldmeter.info).^5^

PE has a negative effect on quality of life, which extends beyond sexual dysfunction. Despite these serious psychological consequences of PE, only a few men seek treatment. Language is an important barrier both for patients and physicians involved in the management of PE. Patients are more comfortable in explaining their condition when they can express in their own language. Also, the availability of diagnostic tools in local languages allow for quantification and follow-up. We aimed to develop a validated translation of PEDT to be used in the Urdu speaking population. Urdu being a living language, spoken by almost a 100 million people around the world^6^ (ORGI, 2011).

## METHODS

This Cross-Sectional study was conducted at the urology section of the Aga Khan University Hospital, Karachi, for six months duration i.e., from July 2018 to December 2018 after institutional and ethical review committee approval (ERC Number: 5373-Sur-ERC-18, Dated: 6^th^ June, 2018). The translation was performed using a multi-step process described by WHO.^7^ PEDT (original English version) was translated into Urdu by two experienced urologists (native URDU speaking), individually, with more than five years of clinical experience to diagnose and treat men with sexual dysfunction, including PE. Combining the two translations, a single Urdu version of PEDT was developed, which was further discussed with the panel of experts and modified as suggested. This initial Urdu version was then translated into English by a bilingual linguist and the final Urdu version of PEDT was developed, by comparing it with the original English PEDT version.

All patients with age 20 to 50 years, who were in a stable sexual relationship (heterosexual) for a minimum duration of six months, visiting the urology clinic, that agreed to participate in the study were included. Study participants were given information about the study procedure and informed written consent was obtained. Study participants filled the PEDT (Urdu) before and after consultation. A pilot study was conducted before the actual validation, including 20 participants (10 with PE and 10 without PE), using process described by WHO^11^. This version was accurate in diagnosing PE and the questions were easy to read and understand by the participants. Data was collected for demographics including age, comorbid conditions, frequency of intercourse, marital status, duration of the relationship, education level, self-reported IELT, type of PE (Lifelong vs acquired). Patients with concomitant erectile dysfunction, on psychiatric medications, major diseases (angina, heart failure, kidney or liver disease, and psychological disorders) and patients with abnormal penile shape were excluded from the study.

SPSS™ v21.0 was used for analyzing the data. Internal consistency, test-retest reliability, and validity was checked by performing psychometric analysis. Cronbach’s alpha ≥ 0.70 was considered to be a significant level of internal consistency.

- Self-estimated IELT and PEDT correlation was evaluated with the Spearman correlation.
- Physician-diagnosed PE and PEDT correlation was evaluated with Spearman correlation.
- Test-retest reliability was evaluated with Pearson correlation by comparing PEDT scores before and after consultation and 0.70 was the minimum acceptable level.

## RESULTS

Initially, 140 eligible participants filled all the questionnaires at the first interview. During the second interview, 32 participants dropped out. In the final study, 61 participants having clinical PE and 47 men without PE were enrolled for validation. Among all patients having PE, 50% (n= 31) had acquired PE. The mean ages of the patients with PE and without PE were 33.77 (±8.70) years and 34.96 (±6.15) years, respectively. Frequency of intercourse was approximately twice weekly in both the groups. Mean age, frequency of intercourse, duration of relationship and level of education were similar in both groups. Mean self-estimated IELT was lower in the PE patients, (1.2 ± 0.52min) as compared to the non-PE patients (3.7 ± 0.91min). (Table 1)

**Table-I:** Demographic profile of the entire cohort.

	Total (n = 108)	Clinical PE(n = 61)	Clinical No PE (n = 47)	p-value
Age (years)	34.9 ± 7.84	33.77 ± 8.70	34.96 ± 6.15	0.429
Frequency of Intercourse (weekly)	2.07± 0.84	1.95 ± 0.87	2.17± 0.86	0.215
Duration of Relationship	7.28 ± 6.02	7.34 ± 6.73	7.20 ± 5.00	0.904
Education [n (%)]				
• Primary school	34 (31.48)	18 (16.66)	16 (14.81)	0.670
• High school	41 (37.96)	25 (23.14)	16 (14.81)	
• Graduate	33 (30.55)	17 (15.74)	16 (14.81)	
Self-reported IELT (minutes)	2.32 ± 1.39	1.18 ± 0.52	3.70 ± 0.91	<0.001
PEDT score Pre-consultation	10.62 ± 4.39	13.39 ± 2.66	7.02 ± 3.51	<0.001
PEDT score Post-consultation	10.29 ± 4.39	13.20 ± 2.58	6.53 ± 3.23	<0.001

Regarding the diagnostic value of Urdu version of PEDT score, we found a negative correlation of 0.6 between the PEDT and self-estimated IELT (p-value <0.001). For PEDT score, using cutoff value of 8 (PE > 8 and no-PE ≤8) there was a false positive rate of 12.8 % (6 men) i.e. PEDT indicated PE, whereas the experts suggested that men did not have clinical PE. There was a false negative rate of 11.5% (7 men) where PEDT score indicated no-PE while the expert diagnosed as clinical PE. Sensitivity and specificity of the Urdu version of PEDT score were 88.5% (95% Confidence interval (CI) = 77.78% -95.26%) and 87.23 % (95% CI = 74.26%-95.17%) respectively (Table-II).

**Table-II:** Sensitivity and Specificity of PEDT scores of greater/lesser than 8 with clinical diagnosis of PE.

	Physician Diagnosis PE % (n)	Physician Diagnosis No PE % (n)	Total
PEDT >8	88.52 % (54)	12.76 % (6)	60
PEDT 8 or less	11.48 % (7)	87.23 % (41)	48

Total	n = 61	n = 47	

There was excellent internal consistency in Urdu version of PEDT, calculated as 0.93 Cronbach’s alpha score (95% CI = 0.905 - 0.948). The test-retest reliability for each item was evaluated with Pearson correlation and found to be significant for all five items and 0.941 (P-value < 0.001) was the correlation coefficient of the total PEDT score (Table-III). Moreover, mean PEDT scores pre-consultation and post-consultation were similar (13.4±2.66 and 13.2±2.58 respectively) in the PE group. Mean PEDT scores pre-consultation and post-consultation were also similar in the non-PE group (7.0 ±3.50 and 6.53 ±3.23 respectively).

**Table-III:** Test re-test reliability of each question and total PEDT score.

	Question: 1	Question: 2	Question: 3	Question: 4	Question: 5	Total
R	0.948	0.843	0.852	0.919	0.945	0.941
p-value	<0.001	<0.001	<0.001	<0.001	<0.001	<0.001

## DISCUSSON

Premature ejaculation evaluation with PEDT is an excellent tool that helps clinicians in objectively defining and quantifying the condition. PE has a highly variable estimated prevalence worldwide and very limited literature is available from local studies performed on sexual dysfunction. Only few studies done in our country have addressed premature ejaculation. One of the study done in a tertiary care hospital in Abbottabad, reported 13% prevalence of premature ejaculation and it was reported to be the second most common sexual dysfunction.^8^ Other studies from tertiary care hospitals in Pakistan, reported a variable estimated prevalence of PE i.e., 30-34%,^9^ 21% 10and 24-27%.^11^ Various characteristics of PEDT have been validated in other regional languages i.e., Korean,^12^ Persian,^13^ Turkish^14^ and Chinese.^15^ National language of Pakistan is Urdu and easily understood by most people in this region with a population of around 217 million, and a language understood by nearly 100 million. Previously Urdu version of International Index of Erectile Function (IIEF) 16 and Female Sexual Function Index (FSFI) 17 has been validated. This has helped in opening up discussion about sexuality among physicians and patients in the Urdu speaking population. In the initial phase of translation, there was some difficulty in finding easily comprehensible terms in Urdu language for English words used in the original tool. Due to social taboos surrounding sex, there was the scarcity of easily understandable, non-obscene words particularly related to sexual intimacy in Urdu. As it is a private interaction and couples are hesitant in sharing it frankly even among them so words like “ejaculation” and “penetration” are rarely used in formal conversation in Urdu. As PEDT is a self-administered questionnaire, easily comprehensible terms are important to use for a validated questionnaire. We found that patients were able to understand the translation probably due to the context in which it was used and mostly did not require any assistance for filling the proforma. Our results have shown that our translation is a reliable and simple tool for common Urdu speaking people.

Our study showed good internal consistency and excellent stability for the PEDT (Urdu version) on test-retest reliability test than most of the previous published translations in other languages. This may be consequent to the fact that the study was conducted in the urology clinic of a tertiary care private hospital with an active andrology service. In our study there was a strong relationship of the calculated PEDT score for the Urdu version with self-estimated IELT, both for patients with and without PE. This is consistent with the results of Persian and Chinese validation studies of this tool (Huang et al 2014). However, PE cannot be diagnosed with time alone, this is reflected in a relatively lower correlation of 0.6 between IELT and PEDT score. Moreover, there are other factors e.g. situational PE, intermittent symptoms, effect on relationship and self-confidence which need to be taken into account when making a diagnosis of PE, this is shown by some false positive and false negative results when PEDT score was compared with a physician diagnosis of PE.

The five items in PEDT score are closely related, so our study showed good internal consistency of Urdu version of PEDT with Cronbach’s alpha score of 0.93 similar to the study by Pakpour and colleague, for the Iranian version of PEDT, alpha = 0.89.^18^ It is interesting to note that Urdu and Persian are closely related languages in terms of structure and even words used. Correlating the results of the Urdu version of the PEDT questionnaires filled twice, before and after the clinic visit assessed the test-retest reliability and strong correlation was observed. This is a potential limitation as there was a small interval between the two interviews. However, a longer interval between the two interviews can introduce bias in terms of change in symptoms and sometimes by use of treatment including psychosexual counseling by a physician.^19^

Many studies have validated diagnostic tools in the Urdu language (e.g., Urdu translation of the Hamilton Rating Scale for Depression^20^), but none was available for the PEDT in the Urdu language. The strengths of the study are that it is the first to translate and validate PEDT in Urdu and WHO guidelines for translation and adaptation of instruments were followed. Although PE has a variable prevalence but it is underreported worldwide, as admitting having PE is still considered a taboo by many people in our country. There was no objective tool yet to define PE objectively in the Urdu language for doing research work specifically on PE. This would also help the researchers in comparing drugs for PE and will help clinicians to see the results of drugs administered for PE in Urdu speaking patients, by assessing improvement in the PEDT scores.

### Limitations of the study

However, our study is limited in terms of generalizability of results as it is a single-center study conducted at a tertiary care hospital. Nevertheless, with the availability of this validated tool, we can now apply this tool for a wider Urdu speaking population. Our results suggest that the Urdu version of PEDT is a valid tool that can be reliably used to diagnose premature ejaculation objectively among the Urdu-speaking population.

## CONCLUSION

The Urdu translation of PEDT is a valid tool to objectively define PE among Urdu speaking patients easily. It showed adequate internal consistency. This version of PEDT has a good correlation with both self-estimated IELT and clinician diagnosis of PE. To establish it as a routine PE diagnosing tool for Urdu speaking patients, further population-based and multi-center studies are required.

### Authors Contribution:

**MUB:** Did Literature search, data analysis, data interpretation, write-up, and is responsible and accountable for the accuracy or integrity of the work.

**WA:** Conceptualized the study design, data analysis, and data interpretation.

**MS:** Did Data Collection and write-up.

**MHA:** Did Urdu Translation.
